# A critical evaluation of the activity-regulated cytoskeleton-associated protein (Arc/Arg3.1)'s putative role in regulating dendritic plasticity, cognitive processes, and mood in animal models of depression

**DOI:** 10.3389/fnins.2015.00279

**Published:** 2015-08-10

**Authors:** Yan Li, Alan L. Pehrson, Jessica A. Waller, Elena Dale, Connie Sanchez, Maria Gulinello

**Affiliations:** ^1^External Sourcing and Scientific Excellence, Lundbeck Research USA, Inc.Paramus, NJ, USA; ^2^Neuroinflammation Disease Biology Unit, Lundbeck Research USA, Inc.Paramus, NJ, USA; ^3^Behavioral Core Facility, Department of Neuroscience, Albert Einstein College of MedicineBronx, NY, USA

**Keywords:** Arc, Arg3.1, cognition, major depressive disorder, neuroplasticity

## Abstract

Major depressive disorder (MDD) is primarily conceptualized as a mood disorder but cognitive dysfunction is also prevalent, and may limit the daily function of MDD patients. Current theories on MDD highlight disturbances in dendritic plasticity in its pathophysiology, which could conceivably play a role in the production of both MDD-related mood and cognitive symptoms. This paper attempts to review the accumulated knowledge on the basic biology of the activity-regulated cytoskeleton-associated protein (Arc or Arg3.1), its effects on neural plasticity, and how these may be related to mood or cognitive dysfunction in animal models of MDD. On a cellular level, Arc plays an important role in modulating dendritic spine density and remodeling. Arc also has a close, bidirectional relationship with postsynaptic glutamate neurotransmission, since it is stimulated by multiple glutamatergic receptor mechanisms but also modulates α-amino-3-hydroxy-5-methyl-4-isoxazolepropionic acid (AMPA) receptor internalization. The effects on AMPA receptor trafficking are likely related to Arc's ability to modulate phenomena such as long-term potentiation, long-term depression, and synaptic scaling, each of which are important for maintaining proper cognitive function. Chronic stress models of MDD in animals show suppressed Arc expression in the frontal cortex but elevation in the amygdala. Interestingly, cognitive tasks depending on the frontal cortex are generally impaired by chronic stress, while those depending on the amygdala are enhanced, and antidepressant treatments stimulate cortical Arc expression with a timeline that is reminiscent of the treatment efficacy lag observed in the clinic or in preclinical models. However, pharmacological treatments that stimulate regional Arc expression do not universally improve relevant cognitive functions, and this highlights a need to further refine our understanding of Arc on a subcellular and network level.

## Introduction

Cognitive dysfunction is a common aspect of central nervous system diseases that has large implications for patients' daily life function and adds to the socio-economic burden of psychiatric illness. Psychiatric illnesses such as major depressive disorder (MDD) prominently feature disturbances in cognitive domains such as executive function, memory, attention, and psychomotor processing speed that predict functional outcomes (Jaeger et al., 2006; McIntyre et al., 2013). On a theoretical level, proper cognitive function allows for an organism to identify and adapt to changing internal or external environmental demands, and to accrete a repertoire of previously-successful strategies for meeting those demands. Each of these functions require a biological system that is capable of self-reorganization, and thus the machinery governing cellular and dendritic plasticity in the brain can be conceptualized as fundamental processes underlying the biology of cognitive function. Importantly, in addition to a relationship between neuroplasticity and cognitive function, emerging hypotheses on the mechanistic basis for mood dysfunction also prominently feature disturbances in neuroplasticity (Pittenger and Duman, 2008).

In support of these ideas, there is evidence linking altered neural plasticity to clinical populations. For example, MDD patients have reduced hippocampal volume (Saylam et al., 2006; Maller et al., 2007; Nifosì et al., 2010), which has been tied to increased cellular density and reduced neuropil prominence in postmortem tissue (Stockmeier et al., 2004; Cobb et al., 2013). Greater reductions in hippocampal volume among MDD patients are associated with greater illness duration (McKinnon et al., 2009; Travis et al., 2014) and poorer clinical outcome (Frodl et al., 2008; MacQueen et al., 2008). Importantly, successful antidepressant treatment is associated with increased hippocampal volume (Frodl et al., 2008; Schermuly et al., 2011; Tendolkar et al., 2013). Altered hippocampal volumes among MDD patients have been associated with reduced performance on tests of executive function (Schermuly et al., 2011), and memory (Travis et al., 2014; Young et al., 2015). But altered brain volume is not limited to the hippocampus in MDD patients. A recent meta-analysis confirmed that reduced volumes are present in the prefrontal cortex, orbitofrontal cortex and cingulate cortex of depressed patients (Arnone et al., 2012), and there is also evidence for increased amygdala volume in some MDD populations (Saleh et al., 2012).

These volumetric data from human depressed patients are mirrored in non-clinical animal models of depression, which have shown reductions in hippocampal volume after chronic exposure to stressors (Lee et al., 2009) or glucocorticoids (Sousa et al., 1998). Interestingly, in these animal models, the consensus based on unbiased stereological histology data appears to be that the reduced hippocampal volumes are not due to a reduced number of cells, but are rather caused by changes in the neuropil of the affected regions (Müller et al., 2001; Czéh and Lucassen, 2007; Tata and Anderson, 2010). Specifically, chronic exposure to stress or the glucocorticoid corticosterone significantly reduces the length and complexity of apical dendrites in the hippocampus (Woolley et al., 1990; Sousa et al., 2000) or in the prelimbic region of the frontal cortex (Hains et al., 2009). Multiple reports have suggested that these depression models are associated with reductions in the density of dendritic spines or the number of synapses in the hippocampus (Sandi et al., 2003; Tata et al., 2006; Hajszan et al., 2009; Vestergaard-Poulsen et al., 2011) or in the frontal cortex (Hains et al., 2009; Radley et al., 2013). Importantly, these dendritic changes have been associated with impaired cognitive performance (Hains et al., 2009). Based on this line of reasoning, the machinery governing neuronal plasticity can be viewed as candidate targets for interventions aimed at correcting not only more traditional psychiatric symptoms such as mood dysfunction, but also disturbances in cognitive function.

Immediate early genes (IEGs) may represent an entry into understanding the relationship between neuronal plasticity and disturbances in mood or cognitive function. IEGs can be separated into two broad classes—transcription factors, which regulate the expression of other gene transcripts, and effector genes, which can directly modulate cellular processes other than gene transcription (Clayton, 2000). Perhaps the majority of the accumulated knowledge on the relationship between cognitive function and IEG biology has focused on transcription factors such as *c-fos, c-jun*, or *egr-1*. However, more recently an effector IEG known as the activity-related cytoskeleton-associated protein (referred to in this paper as Arc but known alternately as Arg3.1) has garnered an increasing level of interest from the research community, due to its putative relationship to behavioral function, synaptic transmission and dendritic plasticity. This paper seeks to review the accumulated knowledge on the role of Arc in terms of its relationship to neuronal plasticity and cognitive function, particularly as it may relate to MDD.

## Arc expression and its molecular functions in the cell

In this section, we will review information on the basic biology of Arc, its relationship to inter- and intra-cellular signaling, growth factors, and its relationship to long term potentiation (LTP), long term depression (LTD), and synaptic scaling.

### The molecular biology and cellular actions of Arc—a brief overview

The IEG Arc encodes a protein that consists of a coiled-coil domain in the N-terminal region, as well as endocytic protein-binding domains and homology to the cytoskeletal protein spectrin in its C-terminus (Bramham et al., 2010). The spectrin homology domain led to studies examining the role of Arc in regulating the actin cytoskeleton, wherein co-sedimentation analyses revealed an association of Arc with F-actin (Lyford et al., 1995), microtubules, and microtubule-associated protein 2 (MAP2) (Fujimoto et al., 2004). Further studies of Arc's relationship to the actin cytoskeleton revealed that Arc maintains phosphorylation of the actin depolymerization factor cofilin. Therefore, Arc expression acts to preserve the inactive form of cofilin and favors increased F-actin formation (Messaoudi et al., 2007).

Through this close association with F-actin, Arc induces an increase in density of spines and in the proportion of thin and filopodia-like protrusions in hippocampal neurons (Peebles et al., 2010). Expression of mutant Arc unable to interact with the endocytic machinery blocked these effects on spine density and morphology. These *in vitro* findings were corroborated in the hippocampal CA1 and dentate gyrus of Arc knockout (KO) mice, where a reduction in spine density and decreased abundance of thin spines (Peebles et al., 2010) was observed by comparison to wild type mice. Moreover, Arc KO mice had a concomitant increase in mature, mushroom-shaped spines (Peebles et al., 2010), which could indicate that Arc has a negative influence on spine maturation, although this idea is speculative and should be viewed cautiously. Additionally, aberrant Arc expression in the hippocampus in response to chronic N-methyl-D-aspartate (NMDA) receptor hypofunction decreased spine density (Balu and Coyle, 2014). Taken together, these data support a role for Arc in regulating dendritic spine density and morphology.

In addition to its association with F-actin, Arc localizes to postsynaptic density (PSD) 95 and NMDA receptor complexes in the PSD (Husi et al., 2000; Donai et al., 2003; Fujimoto et al., 2004). At synaptic sites, Arc directly interacts with an inactive form of calcium/calmodulin-dependent protein kinase IIβ (CaMKIIβ), and this interaction targets Arc to actin-rich dendritic spines (Okuno et al., 2012). In addition to its association with glutamatergic NMDA receptors, Arc has also been tied to trafficking of the α-amino-3-hydroxy-5-methyl-4-isoxazolepropionic acid (AMPA) receptor, which is thought to be associated with Arc's endocytic protein-binding domains (Chowdhury et al., 2006; Bramham et al., 2010). These data suggest that Arc expression is closely associated with glutamatergic neurotransmission.

Finally, Arc is believed to have functional actions in the cellular nucleus (Ramirez-Amaya et al., 2013), although this aspect of Arc expression is less well-studied than its dendritic functions. Translocation of Arc to the nucleus may regulate transcription and homeostatic plasticity (Korb et al., 2013) by binding to transcriptional regulation sites (Bloomer et al., 2007; Korb et al., 2013), and may be related to Arc modulation of AMPA receptor trafficking (described below).

Thus, Arc expression appears to have a complex set of actions that can regulate the actin cytoskeleton in dendritic regions as well as nuclear transcription factor actions, both of which may be related to glutamatergic neurotransmission. In the following section, we will discuss the relationship between Arc expression and glutamate neurotransmission in further detail.

### The inter-relationship of Arc expression and glutamate neurotransmission

**Figure 1:**
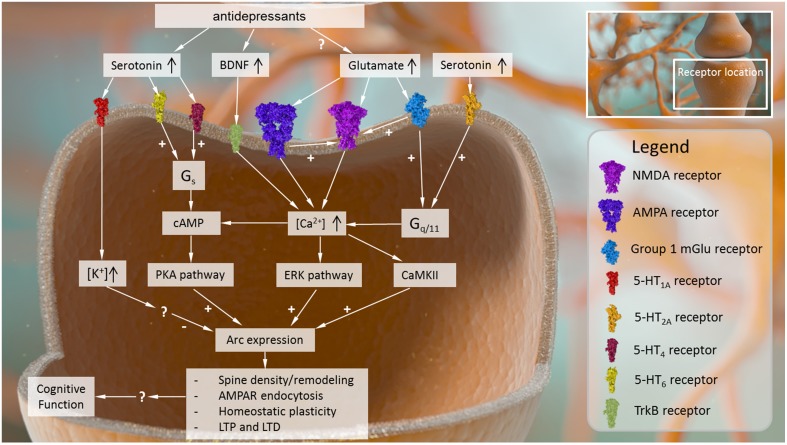
**Intracellular causes and effects of Arc expression in the central nervous system**. Arc expression can be modulated by several converging signaling pathways. Receptor mechanisms that drive up intracellular Ca^2+^ signaling and its downstream sequelae (e.g., AMPA receptors, NMDA receptors, group I metabotropic glutamate receptors, BDNF receptor TrkB) tend to activate Arc expression. Other intracellular signaling cascades related to cyclic AMP may also be capable of stimulating Arc expression, while receptor mechanisms that increase intracellular K^+^ may inhibit Arc expression, although the precise mechanisms driving these effects are not known. Once expressed, Arc plays several roles in modulating neural plasticity, affecting dendritic spine density and remodeling, AMPA receptor surface expression, and processes such as LTP, LTD, and homeostatic plasticity.

The postsynaptic density within the glutamatergic tripartite synapse is associated with multiple interdependent ionotropic and metabotropic glutamate receptor targets that work together to facilitate proper synaptic transmission. This includes the NMDA receptor, which is often conceived as a synaptic coincidence detector (Cull-Candy and Leszkiewicz, 2004), as well as the AMPA/kainate and metabotropic glutamate 5 (mGlu5) receptors, which are key regulators of dendritic membrane depolarization. These glutamate receptor systems are inter-related on multiple levels (see Figure 1). Each of these glutamatergic receptors are thought to be co-localized in postsynaptic excitatory synapses (reviewed in Takumi et al., 1999). Additionally, each of these receptor systems can independently lead to increases in intracellular Ca^2+^ concentrations, either via direct opening of Ca^2+^ channels in the case of NMDA and AMPA/kainate receptors (Pankratov and Lalo, 2014), or via activation of G_q/11_ in the case of mGlu5 receptors (Prothero et al., 1998). Importantly, NMDA receptor function depends critically on activation of AMPA and mGlu5 receptor activation for its function, given that it is both ligand and voltage gated (Foster and Wong, 1987).

Given the interdependence of these receptor systems and the close subcellular localization between Arc and glutamate receptor targets, it should not be surprising that neuronal activation mediated by metabotropic and ionotropic glutamate receptor signaling play central roles in regulating Arc localization throughout the dendritic tree (summarized in Figure 1). For example, following selective activation of excitatory mGlu5 receptors in striatal neuron cultures, there is an increase in Arc mRNA levels and immunoreactivity in cell bodies and dendrites at 1 h post stimulation (Kumar et al., 2012). Importantly, the increase in Arc mRNA expression was inhibited by the administration of calcium channel blockers (Kumar et al., 2012). Moreover, this increased Arc immunoreactivity is dependent on kinases such as CaMKII and ERK, among others, all of which are activated in response to increased Ca^2+^ levels (Kumar et al., 2012). Non-selective stimulation of neuronal cultures with glutamate had the same effect on Arc, and this glutamate-induced elevation in Arc expression was blocked with the mGlu5 receptor negative allosteric modulator MPEP (Kumar et al., 2012).

Further support for a relationship between mGlu5 receptor activation and Arc expression can be found in mice lacking the translational repressor Fragile-X mental retardation protein 1 (FMRP), which display exaggerated mGlu receptor-dependent long-term depression (LTD) and excessive protein synthesis. Arc is a target of FMRP, and *Fmr1* KO neurons express elevated dendritic basal levels of Arc protein, similar to that seen in response to de-phosphorylation of FMRP (Niere et al., 2012). Transduction of a phospho-mimetic mutant of FMRP in *Fmr1* KO hippocampal slices blocks the exaggerated LTD and suppresses basal levels of dendritic Arc (Niere et al., 2012). Thus, following mGlu receptor-LTD, regulation of FMRP is critical for normal protein synthesis and subsequent Arc translation in dendrites where Arc expression is normally suppressed for maintenance of synaptic plasticity and tagging of active synapses (Niere et al., 2012).

Furthermore, NMDA receptor hypofunction in mice deficient in serine racemase (SR), the enzyme that converts L-serine to the NMDA receptor co-agonist D-serine, leads to reduced Arc expression in the hippocampus (Balu and Coyle, 2014). These decreased Arc levels are partially reversed with D-serine treatment (Balu and Coyle, 2014). Conversely, acute D-cycloserine treatment to partially activate NMDA receptors at a dose corresponding to enhanced memory acquisition and consolidation promotes increased Arc protein levels in the dorsal hippocampus 1 h following administration. These elevations in Arc expression were associated with greater activation in CA1 pyramidal neurons, measured in this case as a decreased after-hyperpolarization duration (Donzis and Thompson, 2014). Furthermore, hippocampal Arc expression induced by electroconvulsive shock followed by high frequency stimulation of the perforant pathway could be blocked by local application of either NMDA or AMPA receptor antagonists (Steward and Worley, 2001). In addition, there is a NMDA receptor-dependent increase in Arc mRNA and protein levels in the CA1 region of the hippocampus following a fear learning paradigm in novel contextual environments (Tayler et al., 2011). Thus, it appears that Arc expression is critically dependent on the function of stimulatory postsynaptic metabotropic and ionotropic glutamate receptors.

However, the relationship between glutamate neurotransmission and Arc expression is not unidirectional. There are several lines of evidence indicating that Arc expression can also modulate the efficacy of postsynaptic glutamate neurotransmission. For example, following mGlu5 receptor activation, Arc acts downstream of the myocyte-enhancer factor 2 (MEF2) to decrease excitatory postsynaptic potentials (EPSPs; Wilkerson et al., 2014). In cultures from Arc KO mice, the inhibitory effect of MEF2 on evoked EPSCs is abrogated, and Arc overexpression in these cultures restores the decreased EPSCs (Wilkerson et al., 2014). Thus, it appears that increase of Arc expression can reduce the postsynaptic consequences of mGlu5 receptor stimulation. This idea is further supported by evidence that increased synaptic Arc localization in one study was inversely correlated with surface GluA1 expression (Okuno et al., 2012).

Moreover, it appears that Arc's nuclear expression mentioned above may play an important role in modulating glutamate receptor expression, and has a different time course than its cytoplasmic effects. While cytoplasmic or dendritic Arc expression happens over a relatively short time frame, transport to the nucleus increases progressively after neuronal stimulation. Following depolarization with the GABA_A_ receptor antagonist bicuculline, there is a large increase in expression of nuclear Arc that is maximal 8 h after activation (Korb et al., 2013) and is blocked with inhibitors of the MEK-ERK pathway. Early evidence suggests that nuclear, not cytoplasmic, Arc expression promotes a decrease in surface GluA1 expression and corresponding decrease in mEPSC amplitude and synaptic strength, given that this effect is impaired by preventing nuclear transport of Arc (Korb et al., 2013). Moreover, in the nucleus, Arc induction associates with promyelocytic leukemia nuclear bodies (PMLs; Korb et al., 2013). This association with PMLs is thought to mediate Arc's effects on GluA1, based on evidence that disrupting PML expression blocks Arc's ability to decrease surface GluA1 expression, while ectopic PML expression mimics the effect of Arc on GluA1 (Korb et al., 2013). Thus, over longer periods following neuronal activation, in contrast to brief time points that promote cytoplasmic Arc expression, there is an increase in nuclear Arc that acts to modulate synaptic scaling and promote homeostatic plasticity (described in detail below).

Taken together, these data suggest that Arc expression has a dual role in the regulation of synaptic strength, with a transient effect involved in creating new dendritic spines followed by a slower, longer lasting role that tends to reduce the postsynaptic consequences of glutamatergic neurotransmission.

### The roles of Arc in LTP and LTD formation and homeostatic plasticity

Several forms of synaptic plasticity, such as long-term potentiation (LTP) and long-term depression (LTD) are thought to support memory formation and learning (Turrigiano and Nelson, 2000; Escobar and Derrick, 2007). This also includes the more recently discovered homeostatic plasticity (also known as synaptic scaling), a phenomenon which describes the ability of neurons to adjust the relative strength of their synapses within a range that allows for further increases or decreases in synaptic transmission (Turrigiano and Nelson, 2000; Davis, 2006). Notably, Arc is involved in the regulation of all of these types of plasticity, especially in the hippocampus and cortex (reviewed in Tzingounis and Nicoll, 2006; Bramham et al., 2010; Shepherd and Bear, 2011; Steward et al., 2015). In 2000, Guzowski et al. first showed that infusion of Arc antisense oligonucleotides in the hippocampus impairs the maintenance, but not induction of LTP in the dentate gyrus (Guzowski et al., 2000). Consistent with these electrophysiology results, rats infused with Arc antisense oligonucleotides had deficits in memory consolidation, but had normal memory acquisition in a spatial water maze (Guzowski et al., 2000). These findings were confirmed by Messaoudi et al. (2007) who showed that Arc mRNA is upregulated during hippocampal LTP (Messaoudi et al., 2007). Infusions of Arc antisense oligonucleotides 2 h after LTP induction disrupted changes in the actin cytoskeleton and inhibited LTP (Messaoudi et al., 2007). In addition, Arc knockout (KO) mice exhibit deficits in the late phase of LTP (Plath et al., 2006), although the early phase of LTP is enhanced in these animals (Plath et al., 2006). This effect may be due to deficits in homeostatic plasticity that might leave synapses in a constitutively more plastic state and thus increase short-term plasticity (Shepherd and Bear, 2011).

Arc also plays a role in the regulation of the activity-dependent depression of synaptic transmission. In addition to deficits in LTP, Arc KO mice have impaired hippocampal LTD induced by the stimulation of NMDA or metabotropic glutamate receptors (Plath et al., 2006; Park et al., 2008). Moreover, impairment of Arc expression with antisense oligonucleotides blocks the late phase of mGlu receptor-dependent LTD (mGluR-LTD; Waung et al., 2008). Finally, Arc induced by the exposure of animals to novelty makes hippocampal neurons more susceptible to mGluR-LTD (Jakkamsetti et al., 2013).

LTD requires rapid protein synthesis and internalization of surface glutamate receptors (Man et al., 2000; Snyder et al., 2001; Waung et al., 2008). As discussed above, Arc plays a role in internalizing AMPA receptors by interacting with dynamin and endophilin 2/3 (Chowdhury et al., 2006). Accordingly, cultured neurons from Arc knockout mice have increased AMPA receptor surface expression, increased spontaneous glutamate transmission and exhibit a decreased rate of AMPA receptor endocytosis (Shepherd et al., 2006). In contrast, neurons overexpressing EGFP-labeled Arc transgene in organotypic hippocampal brain slices have reduced AMPA receptor surface expression and smaller AMPA receptor currents, but regular NMDA receptor currents (Rial Verde et al., 2006). Furthermore, synthesis of Arc during LTD increases endocytosis of AMPA receptors (Waung et al., 2008). Taken together, these studies provide strong evidence that Arc decreases surface expression of AMPA receptors. However, these results should be interpreted with caution as they were obtained with cultured neurons or cultured brain slices taken from neonatal animals and might not be translatable to the adult *in vivo* conditions. For instance, in acute hippocampal slices prepared from older Arc KO mice, there are no deficits in AMPA receptor-dependent basal glutamate transmission (Plath et al., 2006). There is also no rundown of AMPA receptor responses in the dentate gyrus of anesthetized rats after induction of Arc with electroconvulsive shock (Steward et al., 2015). Therefore, alternative signaling pathways involved in AMPA receptor trafficking might exist *in vivo* that are able to compensate the effect of Arc on AMPA receptor endocytosis.

In addition to its role in LTP and LTD, Arc is involved in homeostatic plasticity (Rial Verde et al., 2006; Shepherd et al., 2006; Wang et al., 2006; Shepherd and Bear, 2011). Current theory suggests that this type of plasticity is needed to counteract possible over-excitation or silence of synapses due to global changes in network activity and/or Hebbian plasticity (Davis, 2006). In cortical or hippocampal neuronal cultures, prolonged blockade of neuronal activity with the sodium channel blocker tetrotoxin (TTX) leads to widespread enhancement of spontaneous glutamatergic transmission (Turrigiano et al., 1998; Shepherd et al., 2006). Interestingly, treatment with TTX down regulates Arc protein levels in these neurons (Shepherd et al., 2006). Moreover, Arc overexpression is able to block the TTX-induced up regulation of synaptic transmission (Shepherd et al., 2006). Synaptic upscaling in response to TTX is also blocked in cultured neurons from Arc KO mice (Shepherd et al., 2006).

Thus, Arc is involved in several forms of synaptic plasticity, including LTP, LTD and homeostatic plasticity, that rely on remodeling of dendritic spines and changes in AMPA receptor trafficking. Downstream signaling cascades involved in the distinct effects of Arc in each form of synaptic plasticity, and especially the ones engaged during LTP, still remain to be elucidated.

### The relationship between Arc, BDNF, and LTP

In addition to its relationship to glutamate neurotransmission, Arc functions seem to be closely related to those of growth factors, such as brain-derived neurotrophic factor (BDNF). BDNF signaling is thought to be mediated through the tropomyosin-receptor kinase (Trk) B receptor (Soppet et al., 1991), and can have multiple effects on intracellular signaling cascades including increases in intracellular Ca^2+^ concentrations (Berninger et al., 1993; Marsh and Palfrey, 1996; Nakata and Nakamura, 2007). Thus, BDNF-TrkB signaling seems to share some intracellular Ca^2+^-mediated signaling pathways with excitatory glutamatergic receptors such as AMPA, NMDA, and group I mGlu receptors.

Given this information, it is perhaps not surprising to find that BDNF can increase Arc expression, a phenomenon that has been observed in cultured neurons (Yasuda et al., 2007), in synaptoneurosome preparations (Yin et al., 2002), or *in vivo* in the cortex (Benekareddy et al., 2013) or dentate gyrus (Wibrand et al., 2006) of rodents. But the close relationship between BDNF-TrkB signaling and Arc does not stop there. Recent empirical data has demonstrated that intracranial injections of BDNF cause a rapid increase in the surface expression of AMPA receptors (Li and Wolf, 2011; Reimers et al., 2014), and over longer periods of time mediates a reduction in AMPA receptor surface expression (Reimers et al., 2014) that resembles Arc's role in synaptic scaling. Furthermore, BDNF appears to be closely related to LTP, given evidence that *in vivo* BDNF infusions can induce LTP (Messaoudi et al., 1998), that LTP is impaired in BDNF KO mice (Patterson et al., 1996), and that electrical stimulation patterns capable of inducing LTP also stimulate BDNF release (Patterson et al., 1992). Thus, not only is Arc inducible by BDNF, but they appear to modulate similar aspects of postsynaptic glutamate neurotransmission.

Furthermore, recent evidence suggests that synaptic consolidation at excitatory medial perforant path-granule cell synapses requires BDNF signaling and Arc induction (Bramham, 2007). By modulating the spatial and temporal translation of newly induced Arc protein and constitutively expressed Arc mRNA in neuronal dendrites, BDNF may effectively control the window of synaptic consolidation (Soulé et al., 2006). This notion is supported by the observation that Arc-dependent synaptic consolidation in dentate gyrus is activated in response to brief local infusion of BDNF in rats (Messaoudi et al., 2007). Consistent with the related molecular mechanism, recovery of memory acquisition impairment in cholinergic lesioned rats was accompanied by normalization of Arc and BDNF levels in the hippocampus (Gil-Bea et al., 2011). These findings support the idea that Arc plays a role in BDNF-induced neuroplasticity, as well as some aspects of cognitive function.

Interestingly, there is also evidence supporting a role for BDNF in mood disorder pathophysiology. For example, there is substantial evidence that BDNF and TrkB receptor levels are reduced in rodent models of MDD induced by chronic stress (Smith et al., 1995; Ueyama et al., 1997). Additionally, antidepressant treatments increase BDNF levels, whether in rodent models of MDD, or in depressed patients (Chen et al., 2001; Duman and Monteggia, 2006; Kozisek et al., 2008; Autry et al., 2011). Therefore, given the apparently close relationship between Arc and BDNF, it is plausible to expect that Arc expression may also be altered in depression, and respond to antidepressant treatments. Thus, in the following section we will review data on the relationship between stress and Arc expression, and behavioral measurements of cognitive function or mood.

## Arc in MDD-related animal models

Stress is considered a major contributing factor in MDD, primarily based on consistent evidence that high levels of stress confer a greater risk for developing MDD (e.g., Wurtman, 2005; Melchior et al., 2007; Sheets and Craighead, 2014). As a result, most rodent models of MDD prominently feature stress as a method of inducing depression-like changes in behavior or CNS biology. Given the putative relationship between MDD, altered neuroplasticity and Arc expression, it seems logical to examine how Arc expression is altered in stress models and how these alterations may be related to the pathology underlying MDD or to cognitive function.

### Stress effects on Arc expression

Although modern theory typically emphasizes a role for chronic stress in the development of MDD, the majority of animal studies that have examined the relationship of stress to Arc expression have used acute stressors, and further have tended to focus on the frontal cortex. By far the most common stressor used in this literature has been acute restraint stress, and although there is substantial variation in the specific methods, there is a consensus that acute restraint stress induces a significant increase in Arc gene or protein expression in the frontal cortex (see Table 1; Mikkelsen and Larsen, 2006; Molteni et al., 2010; Drouet et al., 2015), and more specifically in the prelimbic, infralimbic (Ons et al., 2004, 2010; Trnecková et al., 2007), and anterior cingulate PFC subregions (Ons et al., 2004, 2010). Moreover, this phenomenon appears to be mediated by glucocorticoid receptors (GRs), given that it does not occur in GR knockout mice (Molteni et al., 2010). Furthermore, the acute stress induced increase in Arc does not appear to be selective to acute restraint stress, as acute predator scent stress also significantly upregulated Arc expression in the frontal cortex and its subregions (Schiltz et al., 2007; Kozlovsky et al., 2008).

**Table 1:** **Stress effects on Arc expression**.

**Species**	**Sex**	**Stressor**	**Arc**	**Brain regions**	**References**
Rat	M	30 min restraint stress	Increased	PL, IL, MA	Trnecková et al., 2007
Rat	M	30 min restraint stress	Increased	mPFC	Mikkelsen and Larsen, 2006
Rat	M	30 min restraint stress	Increased	CC, PL, IL, PIR, LS, MA	Ons et al., 2004
Rat	M	1 h immobilization stress	Increased	CC, PL, IL, PIR, LS, MA	Ons et al., 2010
Rat	M	1 h restraint stress/10 min open field	Increased	PFC	Drouet et al., 2015
Mouse	M	1 h restraint stress	Increased	FC, HC	Molteni et al., 2010
Rat	M	1 h restraint stress	Increased	PL, IL, MA	Trnecková et al., 2007
Rat	M	2 h restraint stress	Increased	PL, IL	Trnecková et al., 2007
Rat	M	2 h restraint stress	Increased	Cx	Benekareddy et al., 2013
Rat	M	2 h restraint stress	No effect	CA1, CA3, DG	Benekareddy et al., 2013
Rat	M	4 h restraint stress	Increased	PL, IL	Trnecková et al., 2007
Rat	M	acute predator scent stress: 30 min post stress	Increased	FC, CA1	Kozlovsky et al., 2008
Rat	M	acute predator scent stress: 7 days post stress	Increased	FC, CA1, CA3, DG	Kozlovsky et al., 2008
Rat	M	acute predator stress	Increased	PL, IL, VO, LO	Schiltz et al., 2007
Rat	M	Chronic corticosterone po (50 μg/mL 14 days, 6 days titrate off) + washout (2–3 weeks)	Increased	LA	Monsey et al., 2014
Mouse	M	Chronic unpredictable stress 3 weeks	Increased	HC	Boulle et al., 2014
Mouse	M	Chronic mild stress	Reduced	FC, CC, CA1	Elizalde et al., 2010
Rat	M	11 days 1 h immobilization stress	Reduced	CC, PL, IL	Ons et al., 2010

*The available data generally suggests that acute stress enhances Arc expression in a variety of brain regions, while chronic stress has more complex and regionally-dependent effects on Arc expression. CA1, Cornu Ammonis area 1; CA3, Cornu Ammonis area 3; CC, cingulate cortex; Cx, Cortex; DG, dentate gyrus; FC, frontal cortex; HC, hippocampus; IL, infralimbic cortex; LA, lateral amygdala; LO, lateral orbital cortex; LS, lateral septum; MA, medial amygdala; mPFC, medial prefrontal cortex; PFC, prefrontal cortex; PIR, piriform cortex; PL, prelimbic cortex; VO, ventral orbital cortex*.

Importantly, Yuen et al. (2009), demonstrated that acute stressors of various types including restraint stress induced a significant increase in AMPA receptor expression that was associated with increases in cortical AMPA and NMDA receptor-dependent EPSCs. This increase lasted for at least 24 h and was mediated via glucocorticoid receptors (GRs) in cortical pyramidal cells. The acute stress-induced increases in AMPA receptor-mediated EPSCs has been replicated by the same group several times (Yuen et al., 2011, 2012), and in addition the same research group observed increases in the firing rate of PFC pyramidal neurons after acute stress (Yuen et al., 2013). Thus, it seems clear that acute stress is associated with an increase in glutamatergic neurotransmission. Given the relationship between glutamate receptor activation and Arc expression, as well as data suggesting that stress-induced Arc expression depends on GRs, it is likely that the increase in Arc expression observed in the frontal cortex is secondary to a GR-mediated increase in glutamate neurotransmission.

In addition to acute stress-induced increases in PFC Arc expression, there appears to be a consensus that acute stress up-regulates Arc expression in the medial amygdala (Ons et al., 2004, 2010; Trnecková et al., 2007). However, within the hippocampal formation, data on the effects of acute stress on Arc expression are equivocal, with some research groups showing increases (Kozlovsky et al., 2008; Molteni et al., 2010), and others showing constitutive hippocampal Arc expression, but no stress-induced changes (Ons et al., 2004, 2010).

In the context of chronic or repeated stress administration, there are far fewer studies of Arc expression (Table 1). Those that are available suggest that Arc expression in the frontal cortex is reduced after repeated stress (Elizalde et al., 2010; Ons et al., 2010), increased in the lateral (Monsey et al., 2014) and medial amygdala (Ons et al., 2010), and increased in the lateral septum (Ons et al., 2010). We only identified two studies that examined the effects of chronic stress on hippocampal Arc expression, and once again the results are equivocal, with one study showing a significant increase (Boulle et al., 2014) and another showing a significant decrease (Elizalde et al., 2010).

In general, more replications of these results are needed in order to understand how chronic stress alters this aspect of neuroplasticity. However, based on the available data there are some early trends that seem to be present. Specifically, Arc expression in the PFC appears to be stimulated by acute stress but inhibited by chronic stress, while Arc expression in the amygdala seems to be stimulated by stress regardless of its chronicity. Importantly, there is no clear trend in terms of how either acute or chronic stress alters Arc expression in the hippocampus. These data may be particularly interesting from the perspective that human MDD populations have reduced cortical volume (Arnone et al., 2012) and may also have increased volume in the amygdala (Saleh et al., 2012), which on the surface seems to mirror the regional effects chronic stress has on Arc expression.

Another important trend seems to be that stress-induced changes in Arc expression mirror changes in glutamate neurotransmission. Within the cortex, acute stress was associated with an increase in Arc expression, as well as increases in AMPA receptor expression, AMPA-mediated EPSCs and pyramidal neuron firing. However, chronic stress appears to induce an opposite response. Yuen et al. (2012) demonstrated that repeated stressors significantly reduce AMPA-mediated EPSCs in cortical pyramidal neurons and significantly reduced the surface expression of AMPA receptors. Additionally, Yuen et al. (2013) demonstrated that repeated stress significantly reduced the firing rate of cortical pyramidal neurons. These reductions in glutamate neurotransmission could provide an explanation for the lack of Arc induction in the cortex by chronic stressors. However, it is important to note the possibility that Arc itself may play a role in some of the reduced glutamate neurotransmission induced by repeated or chronic stressors. Repeated stimulation of Arc expression, particularly within the nuclear cellular compartment, could theoretically play a role in the reduced surface AMPA receptor expression, reduced AMPA-mediated EPSCs, and reduced pyramidal cell firing observed after chronic stress. Thus, Arc-dependent AMPA trafficking may represent an example of a normal synaptic plasticity mechanism that becomes maladaptive after chronic stress, although it is important to note that this idea has yet to be empirically tested.

### Stress effects on mood

The relationship between stress and development of mood disorders is complex. While there is sufficient evidence suggesting a link between stressful life events and depression in patients (Brown et al., 1987; Strauss et al., 2011; Tao et al., 2011; Baarendse and Vanderschuren, 2012), it is difficult to establish a causal relationship. Some studies support an alternative theory, that events are perceived to be stressful by depressed patients (Liu and Alloy, 2010; Hamilton et al., 2013). In rodent models, chronic stress manipulations (such as chronic mild stress, chronic social stress, and repeated single stress) more reliably induce depression-like behavior (increased immobility in forced swim test or tail suspension test, and reduced preference for sweetened water-anhedonia), at least in a subset of animals. Yet some studies report no change in depression-like behavior after applying chronic stressors. On the other hand, acute stresses are less effective in inducing depression-like behavior in rodents (for more in-depth discussion, see reviews Anisman and Matheson, 2005; Sickmann et al., 2014). As chronic stress induces imbalance of Arc expression between cortex and amygdala (as discussed in previous section), these rodent behavioral readouts are consistent with the hypothesis that dysregulation of Arc or neuroplasticity is involved in mood disorders.

### Stress effects on cognitive function

Given the putative relationship between Arc expression and neuroplasticity, it seems interesting to investigate how acute and chronic stressors alter plasticity-dependent forms of cognitive function, such as learning and memory. Unfortunately, there is a substantial mismatch between the primary methods used to investigate stress-induced changes in Arc expression and those used to investigate stress-induced changes in cognitive function. For example, while most labs have used acute stressors to investigate Arc function, the majority of labs focused on stress-induced changes in cognitive function have used chronic stressors (Table 2). Additionally, while the hippocampus has not been a primary focus in studies investigating stress-induced changes in Arc expression, most labs have investigated stress-induced cognitive changes using hippocampus-dependent cognition tasks.

**Table 2:** **Stress effects on cognitive function**.

**Species**	**Sex**	**Stressor**	**Task**	**Effect**	**References**
Rat	M	1 or 4 h restraint stress	NOR	Impairment	Vargas-Lopez et al., 2015
Mouse	M	1.5 h restraint stress	NOR	Impairment	Guercio et al., 2014
Rat	M	20 min restraint stress	MWM	Impairment	Kasar et al., 2009
Rat	M	30 min restraint stress acute	MWM	Improvement	Zheng et al., 2007
Rat	M	3 h restraint before water maze probe trial	MWM	Impairment	Buechel et al., 2014
Rat	M	30 min acute stress	AST	Improvement	Thai et al., 2013
Rat	M	2.5 h restraint stress	SA	Impairment	Amin et al., 2014
Rat	M	2 h restraint stress	FCO	Improvement	Cordero et al., 2003
Rat	M	6 h restraint stress for 7 days	NOR	Impairment	Bowman et al., 2009
Rat	M	1 h restraint stress for 10 days	NOP	Impairment	Gomez et al., 2012
Rat	M	6 h restraint stress for 14 days	NOP	Impairment	Park et al., 2015
Rat	F	6 h restraint stress for 7 days	NOP	Improvement	Bowman et al., 2009
Rat	M	6 h restraint stress 7 days	NOR	Impairment	Bowman et al., 2009
Rat	M	6 h restraint stress 13 days	RAM	Improvement	Bowman et al., 2009
Rat	F	6 h restraint stress 21 days	RAM	Improvement	Bowman et al., 2009
Rat	M	6 h restraint stress 21 days	RAM	Impairment	Bowman et al., 2009
Rat	F	Chronic unpredictable restraint 21 days	RAWM	No effect	Ortiz et al., 2015
Rat	M	Chronic unpredictable restraint 21 days	RAWM	Impairment	Ortiz et al., 2015
Rat	M	5 mg/kg s.c. corticosterone 21 days	MWM	Impairment	Trofimiuk and Braszko, 2015
Mouse	M	8 h restraint stress 21 days	MWM	Impairment	Tian et al., 2013
Rat	M	6 h restraint for 21 days	MWM	Impairment	Kasar et al., 2009
Mouse	M	2 h restraint stress daily for 8 weeks	MWM	Impairment	Huang et al., 2015
Mouse	M	Chronic unpredictable stress 28 days	MWM	Impairment	Rinwa and Kumar, 2014
Mouse	M	chronic unpredictable stress 40 days	MWM	Impairment	Bian et al., 2012
Rat	M	5 mg/kg s.c. corticosterone 21 days	BM	Impairment	Trofimiuk and Braszko, 2015
Rat	M	Chronic restraint stress 6 h daily for 21 days	AST	Impairment	Liston et al., 2006
Rat	M	Chronic unpredictable stress 14 days	AST	Impairment	Bondi et al., 2010
Rat	M	Restraint stress 1 h daily for 7 days	AST	Impairment	Nikiforuk and Popik, 2011
Mouse	M	8 h restraint stress 21 days	PA	Impairment	Tian et al., 2013
Rat	M	6 h restraint stress 21 days	FCO	Improvement	Conrad et al., 1999
Rat	M	6 h restraint stress 21 days	FCO	Improvement	Sandi et al., 2001
Rat	M	Chronic corticosterone po (50 μg/mL 14 days, 6 days titrate off) + washout (2–3 weeks)	FCO	Improvement	Monsey et al., 2014

*Stress has complex effects on cognitive function that depend on the chronicity of the stressor, the sex of the experimental subject, and the cognitive task being used. AST, attentional set shifting task; BM, Barnes maze; FCO, fear conditioning; MWM, Morris water maze; NOP, novel object placement; NOR, novel object recognition; PA, Passive avoidance; RAM, radial arm maze; RAWM, radial arm water maze; SA, spontaneous alternation*.

The available data suggest that acute restraint stress induces mixed effects in hippocampus-dependent learning and memory tasks, with most labs showing significant impairments in tasks such as the Morris water maze (Kasar et al., 2009; Buechel et al., 2014) and others showing significant improvements (Zheng et al., 2007). Performance in cognitive tasks that are thought to depend on the extended hippocampal network, such as novel object recognition (Aggleton and Brown, 2005; Barker and Warburton, 2011) are also negatively affected by acute stress (Guercio et al., 2014; Vargas-Lopez et al., 2015). Interestingly, acute stress appears to significantly improve performance in cognitive tasks that depend on the frontal cortex (e.g., attentional set shifting; Thai et al., 2013) or amygdala (e.g., fear conditioning; Cordero et al., 2003), where Arc expression is increased in response to similar stressors (Ons et al., 2010; Monsey et al., 2014).

Chronic stress has some mixed effects on hippocampus-dependent cognitive tasks that may depend on the chronicity of the stressor and on the sex of the experimental subject. Several research groups have demonstrated that repeated restraint stress induces significant impairments in the novel object placement task in male rodents (Bowman et al., 2009; Gomez et al., 2012; Park et al., 2015), while performance in the same task was improved in female subjects under similar stress conditions (Bowman et al., 2009). In a radial arm maze task, performance in male rodents was significantly improved by 6 h restraint stress for 13 days, but impaired by the same stressor over 21 days (Bowman et al., 2009). Additionally, female rodents subjected to 6 h restraint stress per day for 21 days performed significantly better in the same radial arm maze task (Bowman et al., 2009). However, in the Morris water maze and Barnes maze, chronic stress universally seems to impair performance (Kasar et al., 2009; Bian et al., 2012; Tian et al., 2013; Rinwa and Kumar, 2014; Huang et al., 2015; Trofimiuk and Braszko, 2015). Thus, although there are some complexities in terms of how chronic stress modulates performance in hippocampus-dependent cognitive tests, on average stress seems to impair performance, at least in male subjects. Given the greatly increased prevalence of MDD in women, more study of the effects of stress on hippocampus-dependent cognition in female subjects is required. Unfortunately, it is not possible to relate these sex-dependent responses to stress to Arc expression, given that there are no studies that have attempted to investigate sex differences in Arc expression in response to stressful stimuli.

In amygdala-dependent cognitive tasks such as fear conditioning, there is a substantial literature indicating that chronic stress enhances some aspects of fear conditioning acquisition (for example, Conrad et al., 1999; Sandi et al., 2001), and interestingly that it may impair extinction of fear conditioning (e.g., Hoffman et al., 2014), which is thought to be a frontal cortex-mediated cognitive function (Zelinski et al., 2010). Similarly there appears to be a consensus that chronic stress impairs performance in the attentional set shifting task, another frontally-mediated cognitive task (Liston et al., 2006; Bondi et al., 2010; Nikiforuk and Popik, 2011).

These data seem to be in alignment with observations that chronic stressors activate Arc expression in the amygdala while simultaneously suppressing Arc expression in the frontal cortex. Additionally, stress appears to have mixed effects on hippocampus-dependent cognitive tasks, and similarly has mixed effects on Arc expression. Taken together, these data are consistent with the notion that cellular neuroplasticity is critical for stress-related dysregulation of cognitive function.

## Effects of antidepressants on Arc expression

Given the relationship between stress and depression, and the putative dysregulation of neuroplasticity in MDD patients, it has been hypothesized that effective antidepressant treatment requires an ability to modulate cellular neuroplasticity (Duman et al., 1999), which may include Arc expression. Furthermore, if Arc expression is relevant for therapeutic efficacy, then there should be a similar timeframe for the onset of efficacy in mood symptoms and Arc expression. In the following section, we will review the effects of antidepressant drugs on Arc expression in rodents in order to evaluate these ideas. Table 3 presents data on the effects of antidepressant drugs on Arc expression.

**Table 3:** **The effects of antidepressant drugs on Arc expression in rodents**.

**Species/Strain**	**Sex**	**Drug**	**Dose (mg/kg)**	**Route**	**Timing**	**Action**	**Arc**	**Brain Regions**	**References**
Rat/SD	M	Fluoxetine	10	i.p.	Acute	5-HTT inhibitor	–	–	Alme et al., 2007
Rat/SD	M	Paroxetine	5	s.c.	Acute	5-HTT inhibitor	–	–	Castro et al., 2003
Rat/SD	M	Paroxetine	5	i.p.	Acute	5-HTT inhibitor	–	–	Tordera et al., 2003
Rat/SD	M	Paroxetine	5	i.p.	Acute	5-HTT inhibitor	–	–	Pei et al., 2003
Rat/SD	M	Desipramine	5	i.p.	Acute	NET inhibitor	–	–	Pei et al., 2003
Rat/SD	M	Venlafaxine	5	i.p.	Acute	5-HTT, NET inhibitor	–	–	Pei et al., 2003
Rat/SD	M	Venlafaxine	10	i.p.	Acute	5-HTT, NET inhibitor	–	–	Serres et al., 2012
Rat/SD	M	Duloxetine	10	p.o.	Acute	5-HTT, NET inhibitor	Decreased	FC	Molteni et al., 2008
Rat/SD	M	Duloxetine	10	p.o.	Acute	5-HTT, NET inhibitor	Increased	EC, MB	Molteni et al., 2008
Rat/SD	M	Fluoxetine	10	p.o.	4 days	5-HTT inhibitor	–	–	De Foubert et al., 2004
Mouse/C57BL/6J	M	Fluoxetine	20	i.p.	4 days	5-HTT inhibitor	–	–	Ferrés-Coy et al., 2013
Rat/SD	M	Fluoxetine	10	p.o.	1 week	5-HTT inhibitor	–	–	De Foubert et al., 2004
Rat/SD	M	Venlafaxine	10	i.p.	1 week	5-HTT, NET inhibitor	–	–	Serres et al., 2012
Rat/SD	M	Fluoxetine	10	p.o.	2 weeks	5-HTT inhibitor	Increased	CC, OFC	De Foubert et al., 2004
Mouse/C57BL/6J	M	Fluoxetine	20	i.p.	2 weeks	5-HTT inhibitor	Increased	DG	Ferrés-Coy et al., 2013
Rat/SD	M	Paroxetine	5; b.i.d.	i.p.	2 weeks	5-HTT inhibitor	Increased	FC, OFC, PC, CA1	Pei et al., 2003
Rat/SD	M	Desipramine	5; b.i.d.	i.p.	2 weeks	NET inhibitor	Increased	FC, CC, OFC, PC, CA1	Pei et al., 2003
Rat/SD	M	Venlafaxine	5; b.i.d.	i.p.	2 weeks	5-HTT, NET inhibitor	Increased	PC, CA1	Pei et al., 2003
Rat/SD	M	Venlafaxine	10	i.p.	2 weeks	5-HTT, NET inhibitor	Increased	CC, PC	Serres et al., 2012
Rat/SD	M	Fluoxetine	10	i.p.	3 weeks	5-HTT inhibitor	Increased	HC	Alme et al., 2007
Rat/FSL	M	Escitalopram	330 mg drug/kg food	p.o.	3 weeks	5-HTT inhibitor	Increased	CA1, DG	Eriksson et al., 2012
Rat/FSL	M	Nortriptyline	330 mg drug/kg food	p.o.	3 weeks	5-HTT, NET inhibitor	–	–	Eriksson et al., 2012
Rat/SD	M	Duloxetine	10	p.o.	3 weeks	5-HTT, NET inhibitor	Increased	FC, EC, MB	Molteni et al., 2008
Rat/SD	NR	Venlafaxine	10	i.p.	3 weeks	5-HTT, NET inhibitor	Increased	HC	Calabrese et al., 2011
Rat/SD	NR	Venlafaxine	10	i.p.	3 weeks	5-HTT, NET inhibitor	Decreased	FC	Calabrese et al., 2011
Rat/SD	NR	Agomelatine	40	i.p.	3 weeks	Melatonin_1_receptor agonist, melatonin_2_ receptor agonist, 5-HT_2C_ receptor antagonist	Increased	HC	Calabrese et al., 2011
Rat/Wistar	M	Imipramine	20	p.o.	7 weeks	5-HTT, NET inhibitor	Increased	FC, DG	Wibrand et al., 2013
Rat/Wistar	F	Imipramine	20	p.o.	7 weeks	5-HTT, NET inhibitor	Increased	DG	Wibrand et al., 2013

*5-HTT, 5-HT transporter; b.i.d., bis in die; CA1, cornu ammonis area 1; CC, cingulate cortex; DG, dentate gyrus; EC, entorhinal cortex; F, female; FC, frontal cortex; FSL, Flinders Sensitive Line; HC, hippocampus; M, male; MB, midbrain; NET, norepinephrine transporter; NR, not reported; OFC, orbitofrontal cortex; PC, parietal cortex; SD, Sprague Dawley*.

The available data on selective serotonin reuptake inhibitor (SSRI) antidepressants suggests that these drugs upregulate Arc mainly in cortical regions, but also in portions of the hippocampal formation. However, these changes in Arc expression are not seen with short term treatments. For example, De Foubert et al. (2004) found that acute, 4 days, and 7 days administration of fluoxetine (10 mg/kg p.o.) had no effect on Arc expression in the cingulate gyrus, parietal cortex or orbital cortex, whereas 14 days treatment increased Arc mRNA expression in the cingulate and orbital cortices. Similarly, Ferrés-Coy et al. (2013) demonstrated that short term (in this case 4 days) treatment with a very high daily dose of fluoxetine (20 mg/kg i.p.) failed to alter Arc mRNA stimulation in any subregion of the mouse hippocampal formation, but after 15 days fluoxetine significantly increased Arc mRNA expression selectively within the dentate gyrus. Interestingly, inhibition of SERT function via injection of SERT siRNA had an accelerated time course, with increased Arc expression observed after only 4 days of treatment. At this time point, Arc mRNA expression was enhanced in the CA1 as well as the dentate gyrus. Alme et al. (2007) found that acute 10 mg/kg fluoxetine failed to alter Arc expression in the prefrontal cortex, hippocampus and dentate gyrus, but after 21 days Arc expression was significantly increased in the prefrontal cortex and hippocampus. Similarly, several labs have demonstrated that acute treatment with paroxetine had no effects on Arc expression in any region studied, which included the cingulate gyrus, frontal and parietal cortex, striatum and hippocampus (Castro et al., 2003; Pei et al., 2003; Tordera et al., 2003).

Moreover, similar themes are observed with norepinephrine (NE)-centered antidepressant drugs. Chronic dosing (20 mg/kg p.o. for 7 weeks) of the tricyclic antidepressant (TCA) imipramine, a balanced serotonin and NE transporter inhibitor, increased BDNF and Arc mRNA expression significantly in the dentate gyrus in male and female rats, whereas in the prefrontal cortex imipramine only increased BDNF and Arc mRNA expression in male rats (Owens et al., 1997; Wibrand et al., 2013). The TCA desimipramine (5 mg/kg b.i.d. for 2 weeks), which is over 400-fold more selective for the NE transporter than the serotonin (5-HT) transporter (Owens et al., 1997), increased Arc expression in the cingulate gyrus, frontal, orbital, and parietal cortices, as well as the hippocampal CA1 region, whereas there were no effects in the striatum and the dentate gyrus (Pei et al., 2003). However, a study by Eriksson et al. (2012) of nortriptyline, a TCA with approximately 60-fold selectivity for NE transporter over the 5-HT transporter (Owens et al., 1997) in Flinders Sensitive Line rats failed to alter Arc expression in any of the brain regions studied (CA1, DG, parahippocampal region, sensorimotor cortex, and amygdala), although escitalopram treatment significantly elevated Arc expression in the CA1 and DG hippocampal subregions. Since there are no exposure data available for nortriptyline in this study, it is hard to evaluate the impact of these findings. In addition, other antidepressants increased Arc expression after chronic dosing, including serotonin-norepinephrine reuptake inhibitors (SNRI) duloxetine and venlafaxine, and the melatonin/serotonin receptor modulator agomelatine (Molteni et al., 2008; Calabrese et al., 2011; Serres et al., 2012).

Taken together, these data suggest that Arc expression is upregulated by treatment with monoamine-centered antidepressants. The increase in Arc expression induced by these treatments generally required chronic administration before any effect on Arc expression is observed, which appears to mimic the therapeutic lag observed with these compounds in the clinic. Interestingly, treatments that have putatively fast-acting antidepressant efficacy, such as ketamine administration and electroconvulsive therapy also acutely increase Arc expression (Larsen et al., 2005; Dyrvig et al., 2012; de Bartolomeis et al., 2013). Thus, induction of cortical Arc expression may have use as a heuristic for onset of antidepressant therapeutic efficacy in preclinical models of MDD, although more studies are needed to develop and fully evaluate this idea.

## Effects of selective serotonin receptor mechanisms on Arc expression

The requirement for chronic SSRI treatment in order to induce Arc expression could indicate that desensitization of some serotonergic receptor mechanisms, e.g., 5-HT_1A_ receptors, is required in order to effectively stimulate Arc expression. This idea is supported by the observation that although acute paroxetine administration at 5 mg/kg s.c. does not alter Arc expression, acute paroxetine in combination with the selective 5-HT_1A_ receptor antagonist WAY-100635 significantly increased Arc mRNA expression in the cingulate gyrus, as well as the frontal and parietal cortices without altering Arc stimulation in the striatum or in the CA1 subregion of the hippocampal formation (Castro et al., 2003). The ability of WAY-100635 as well as other 5-HT_1A_ receptor antagonists such as NAD-299 to increase paroxetine-induced Arc expression was replicated in a separate study (Tordera et al., 2003), although the Arc stimulatory effects observed by Castro et al. (2003) were not replicated in every brain region.

One potential explanation for these data could be that 5-HT_1A_ receptor activation secondary to 5-HT transporter inhibition reduces the firing rate of raphe serotonergic neurons, thereby limiting extracellular 5-HT output and reducing the ability of the serotonergic system to activate other serotonergic targets. In order to understand this idea further, the following section will review the available data on the effects of selective 5-HT receptor ligands on Arc expression and discuss the relevance of these receptor targets for cognitive function. Unfortunately, there is limited or no information in the literature for most 5-HT receptor subtypes, and mainly the role of 5-HT_1A_ and 5-HT_2_ receptor-mediated effects has been subject to investigation. The relationship between 5-HT receptor activation and Arc expression is represented in Figure 1.

### 5-HT_1A_ receptors

5-HT_1A_ receptors are inhibitory serotonergic receptor targets that can operate through a variety of mechanisms. Perhaps the most well-understood function of the 5-HT_1A_ receptor is as an inhibitory G-protein coupled autoreceptor expressed on serotonergic cell bodies of the brainstem raphe nuclei. Activation of these autoreceptors limits the firing rate of these cells and therefore also the amount of 5-HT released in target regions (Blier and Ward, 2003). However, it is important to consider that 5-HT_1A_ receptors do not exist uniformly as serotonergic autoreceptors. A subset of 5-HT_1A_ receptors also exist as postsynaptic heteroreceptors, which have fast inhibitory effects mediated by inwardly rectifying potassium channels (reviewed in Pehrson and Sanchez, 2015). Given the fact that 5-HT_1A_ heteroreceptors are present in glutamatergic pyramidal cells and GABAergic interneurons in the cortex and hippocampus, as well as the complex inter-relationship between these cell types in the cortical and hippocampal circuits (reviewed in Pehrson and Sanchez, 2014, 2015), activation of 5-HT_1A_ heteroreceptors can be expected to produce mixed excitatory and inhibitory effects on glutamatergic neurotransmission (for example, see Llado-Pelfort et al., 2012b).

Selective 5-HT_1A_ receptor activation induces a small but significant increase in Arc expression in the medial prefrontal cortex (Bruins Slot et al., 2009), a region where 5-HT_1A_ receptors can be found on both GABAergic interneurons and pyramidal cells (Celada et al., 2013), without having an effect on striatal Arc expression. Moreover, the absence of 5-HT_1A_ receptor-stimulated Arc expression in the striatum may be expected, given the relative absence of this receptor in the striatum (Aznar et al., 2003), and the mixed excitatory and inhibitory effects 5-HT_1A_ receptor agonists have in striatal input regions such as the cortex (Llado-Pelfort et al., 2012b). Data on the effects of 5-HT_1A_ receptor antagonism on Arc expression is somewhat contradictory. At least two groups have demonstrated that WAY-100635 has no effect on Arc expression alone in the cortex or hippocampus (Castro et al., 2003; Tordera et al., 2003). However, the 5-HT_1A_ receptor antagonist NAD-299 had equivocal effects in these brain regions. Tordera et al. (2003) found that NAD-299 had no effect on Arc expression in the cortex or hippocampus, while Eriksson et al. (2012) found that NAD-299 significantly increases Arc stimulation in the CA1 and dentate gyrus subregions of the hippocampus.

Thus, it appears that selective 5-HT_1A_ receptor modulation in either direction may have some limited and regionally-dependent effects on Arc expression. However, if one considers the information that 5-HT_1A_receptor antagonism combined with acute SSRI administration increased Arc expression, then there may be two competing hypotheses on the relationship between 5-HT_1A_ receptor activation and Arc expression. On the one hand, stimulation of 5-HT_1A_ autoreceptors in the midbrain raphe nuclei may attenuate the release of 5-HT into the synaptic and extrasynaptic spaces, thereby abrogating the effects of 5-HT on stimulatory serotonergic receptors such as 5-HT_2_, 5-HT_4_, and 5-HT_6_. However, an alternative hypothesis may be that postsynaptic 5-HT_1A_ heteroreceptors directly attenuate the expression of Arc by reducing neuronal depolarization. The fact that acute treatment with SSRI antidepressants can generate significant increases in extracellular 5-HT in multiple brain regions (e.g., Pehrson et al., 2013) while still being unable to drive increases in Arc expression may favor the latter hypothesis.

In terms of cognitive function, the available data suggest that 5-HT_1A_ receptor stimulation has mixed but overall negative effects on cognitive function (Table 4). For example, although low doses of the 5-HT_1A_ receptor agonist 8-hydroxy-2-(di-*n*-propylamino)tetralin (8-OH-DPAT) are neutral in terms of their effects on water maze performance (Riekkinen et al., 1994), while higher doses of 8-OH-DPAT or the 5-HT_1A_ receptor agonist buspirone tend to impair water maze performance (Kant et al., 1998; Meijer et al., 1998). Similarly, 8-OH-DPAT administration impairs performance in novel object recognition (Pitsikas et al., 2005) and in putative spatial working memory tasks such as spontaneous alternation (Seibell et al., 2003; Ulloa et al., 2004). Although it is important to note that there may be sex-dependent effects of 8-OH-DPAT, given that Ulloa et al. (2004) found that this ligand had no effect on spontaneous alternation performance in female rats. It is not known whether these sex differences are due to altered pharmacokinetics or pharmacodynamics, and thus more studies are required.

**Table 4:** **Serotonin receptor effects on cognitive function**.

**Drug**	**Dose (mg/kg)**	**Route**	**Target receptor**	**Action**	**Species**	**Strain**	**Sex**	**Task**	**Effect**	**References**
8-OHDPAT	0.03–0.09	i.p.	5-HT_1A_	Agonist	Rat	Wistar Han	M	MWM	No effect	Riekkinen et al., 1994
8-OHDPAT	0.1, 0.3	s.c.	5-HT_1A_	Agonist	Rat	Wistar	M	MWM	Impairment	Meijer et al., 1998
8-OHDPAT	0.25	i.p.	5-HT_1A_	Agonist	Rat	Sprague Dawley	M	Mod MWM	Impairment	Kant et al., 1998
8-OHDPAT	2	i.p.	5-HT_1A_	Agonist	Rat	Sprague Dawley	M	SA	Impairment	Seibell et al., 2003
8-OHDPAT	0.125–2	i.p.	5-HT_1A_	Agonist	Rat	Sprague Dawley	M	SA	Impairment	Ulloa et al., 2004
8-OHDPAT	0.125–2	i.p.	5-HT_1A_	Agonist	Rat	Sprague Dawley	F	SA	No effect	Ulloa et al., 2004
8-OHDPAT	0.25–0.5	i.p.	5-HT_1A_	Agonist	Rat	Wistar	M	Auto	Impairment	Meneses, 2007
8-OHDPAT	1	i.p.	5-HT_1A_	Agonist	Rat	Wistar	M	Auto	No effect	Meneses, 2007
8-OHDPAT	0.031–0.25	i.p.	5-HT_1A_	Agonist	Rat	Wistar	M	Auto	Improvement	Meneses and Hong, 1999
8-OHDPAT	0.062	i.p.	5-HT_1A_	Agonist	Rat	Wistar	M	Auto	Improvement	Meneses et al., 1997
8-OHDPAT	1	i.p.	5-HT_1A_	Agonist	Rat	Wistar	M	Auto	No effect	Meneses and Hong, 1999
8-OHDPAT	0.1–1	s.c.	5-HT_1A_	Agonist	Rat	Wistar	M	NOR	Impairment	Pitsikas et al., 2005
Buspirone	10	i.p.	5-HT_1A_	Agonist	Rat	Sprague Dawley	M	Mod MWM	Impairment	Kant et al., 1998
S15535	0.16–10	s.c.	5-HT_1A_	Partial agonist	Rat	Wistar	M	SR	Improvement	Millan et al., 2004
S15535	0.16–0.63	s.c.	5-HT_1A_	Partial agonist	Mouse	C57BL/6	M	DNMTS	Improvement	Millan et al., 2004
S15535	1.25–5	p.o.	5-HT_1A_	Partial agonist	Rat	old Wistar	M	DNMTS	Improvement	Millan et al., 2004
WAY100635	0.3–1	i.p.	5-HT_1A_	Antagonist	Rat	CD-COBS	M	NOR	No effect	Pitsikas et al., 2003
WAY100635	0.3–1	s.c.	5-HT_1A_	Antagonist	Rat	Wistar	M	NOR	No effect	Pitsikas et al., 2005
WAY100635	0.001–1	i.p.	5-HT_1A_	Antagonist	Rat	Wistar	M	Auto	No effect	Meneses and Hong, 1999
WAY100135	5–20	i.p.	5-HT_1A_	Antagonist	Rat	Wistar	M	Auto	No effect	Meneses and Hong, 1999
DOI	1	i.p.	5-HT_2A/2C_	Agonist	Rat	Sprague Dawley	M	SA	No effect	Seibell et al., 2003
DOI	0.1–0.25	i.p.	5-HT_2A/2C_	Agonist	Rat	Sprague Dawley	M	Mod MWM	No effect	Kant et al., 1998
DOI	0.1–1	i.p.	5-HT_2A/2C_	Agonist	Rat	Wistar	M	Auto	Impairment	Meneses, 2007
DOI	0.1	i.p.	5-HT_2A/2C_	Agonist	Rat	Wistar	M	Auto	Improvement	Meneses et al., 1997
ketanserin	0.001	i.p.	5-HT_2_	Antagonist	Rat	Wistar	M	Auto	Improvement	Meneses et al., 1997
M100907	0.08	i.p.	5-HT_2A_	Antagonist	Rat	Wistar	M	DALT	No effect	Papakosta et al., 2013
M100907	0.1–3	i.p.	5-HT_2A_	Antagonist	Rat	Wistar	M	Auto	No effect	Meneses et al., 1997
SB242084	0.5	i.p.	5-HT_2C_	Antagonist	Rat	Wistar	M	DALT	No effect	Papakosta et al., 2013

*Auto, autoshaping; DALT, delayed alternation; DNMTS, delayed non-match to sample; F, female; M, male; MWM, Morris water maze; Mod, modified; NOR, novel object recognition; SA, spontaneous alternation; SR, social recognition*.

5-HT_1A_ receptor agonists alter performance in learning and memory tasks such as autoshaping in a seemingly dose-dependent manner. Meneses and colleagues have found that 8-OH-DPAT significantly improves performance in the autoshaping task at doses below 0.25 mg/kg (Meneses et al., 1997; Meneses and Hong, 1999), while doses ranging from 0.25 to 0.5 mg/kg impair performance (Meneses and Hong, 1999; Meneses, 2007), and 1 mg/kg seems to have no effect on autoshaping performance (Meneses and Hong, 1999; Meneses, 2007). Interestingly, 5-HT_1A_ receptor partial agonists such as S15535 seem to have more beneficial effects on cognitive performance. This compound engenders significant improvements in social recognition memory as well as working memory performance in a delayed non-match to sample task (Millan et al., 2004).

When administered alone, 5-HT_1A_ receptor antagonists such as WAY100635 generally have no effect on performance in recognition memory (Pitsikas et al., 2003, 2005), or in autoshaping tasks (Meneses and Hong, 1999).

Moreover, we have not been able to identify any studies that have directly investigated the relationship between Arc stimulation and performance in these cognitive tasks. Additionally, there is a relative paucity of information on the regional effects of selective 5-HT_1A_ receptor agonists on Arc expression, for example in the hippocampus. However, the fact that selective 5-HT_1A_ receptor agonists alone lack the ability to substantially increase cortical Arc expression appears to be in line with the mixed effects this class of ligands have on cortical glutamate neurotransmission (Llado-Pelfort et al., 2012a). This likely reflects the fact that 5-HT_1A_ receptors have a wide-ranging and complex expression pattern featuring opposite directed activities (Celada et al., 2013). Moreover, based on the theory that beneficial effects on learning and memory require stimulation of the neuronal plasticity machinery, and the potentially inhibitory influence 5-HT_1A_ receptors have on Arc expression, it is perhaps unsurprising that 5-HT_1A_ receptor agonists have an overall negative influence on cognitive function.

### 5-HT_2_ receptors

5-HT_2_ receptors are G-protein coupled receptors that have a stimulatory effect on cellular membranes via the activation of G_*q*/11_ and the associated increase in intracellular Ca^2+^. 5-HT_2_ receptors have a relatively strong somatodendritic expression pattern in the neocortex and hippocampus, where they are associated with pyramidal neurons and several subclasses of GABAergic interneuron (Cornea-Hébert et al., 1999; Puig et al., 2010; Bombardi, 2012; Celada et al., 2013). As a result, agonists at 5-HT_2_ receptors such as 2,5-dimethoxy-4-iodoamphetamine (DOI) have mixed effects on glutamate neurotransmission that are regionally-dependent. Within the medial PFC, Puig et al. (2003) demonstrated that acute DOI administration increases the firing rate in approximately 37% of putative pyramidal cells and reduced firing in another 30%, however the overall effect was excitatory (a 2.4-fold increase in population firing over baseline). Within the orbital frontal cortex the overall effect of acute DOI administration was inhibitory (Wood et al., 2012), and similarly 5-HT_2A_ receptors seem to have an overall inhibitory effect in the hippocampus (Shen and Andrade, 1998; Wang and Arvanov, 1998). These differences in the overall effect of DOI administration likely reflect differences in the proportion of pyramidal cells and GABAergic interneurons that express 5-HT_2A_ heteroreceptors.

The effects of 5-HT_2_ receptors are one of the more studied aspects of 5-HT neurotransmission's effects on Arc expression. Pei et al. (2000) demonstrated that combined administration of the monoamine oxidase inhibitor tranylcypromine (5 mg/kg i.p.) and L-tryptophan (100 mg/kg i.p.) increased Arc expression in the cingulate, orbital, frontal and parietal cortices, and the striatum, while reducing Arc expression in the hippocampus. Furthermore, these authors found that cortical Arc mRNA expression induced by this treatment was blocked by the 5-HT_2_ receptor antagonist ketanserin in the cortical regions. However, ketanserin was only able to partially block the effects of the tranylcypromine/L-tryptophan treatment in the striatum and had no effect in the CA1 subregion of the hippocampal formation. Further supporting a role for 5-HT_2A_ receptors in serotonin-stimulated Arc expression, Hirani et al. (2003) found that Arc mRNA expression induced by fenfluramine administration in the frontal and piriform cortices could be blocked by administration of the 5-HT_2A_ receptor selective antagonist M100907 (0.2 mg/kg).

Taken together, these data suggest that cortical Arc mRNA expression is sensitive to 5-HT_2A_ receptor stimulation. This hypothesis was confirmed by Pei et al. (2000), who demonstrated that the 5-HT_2A/2C_ receptor agonist DOI dose-dependently increased cortical Arc mRNA expression, with significant increases in most cortical regions at 1 and 2 mg/kg, and more limited effects at 0.2 mg/kg. Importantly, this effect was blocked by the 5-HT_2_ receptor antagonist ketanserin. The regionally-specific effects of 5-HT_2A_ receptor stimulation are once again demonstrated by the fact that DOI administration only weakly affected Arc mRNA expression in the striatum, and had no effect within the hippocampal formation (Pei et al., 2000). These data were replicated by the same group in a later experiment (Pei et al., 2004). These results were extended by the demonstration that the 5-HT_2A_ receptor selective antagonist M100907, but not the 5-HT_2B/2C_ receptor antagonist SB-206553, was able to reverse the effects of DOI on cortical Arc expression.

Interestingly, this study also demonstrated that DOI-induced Arc mRNA expression could be blocked by a high dose (1 mg/kg) of the non-competitive NMDA receptor antagonist MK-801 or the competitive AMPA receptor antagonist GYKI52466 (25 mg/kg i.p.) These data could suggest that 5-HT_2A_ receptor activation-induced increases in Arc induction are mediated in part by ionotropic glutamate receptors, and is in line with the proposed interrelation between Arc stimulation and glutamate neurotransmission. However, this mechanistic relationship should not necessarily be taken to suggest that 5-HT_2A_ receptor activation-induced increases in Arc stimulation in cortical regions are related to an overall increase in the output of cortical pyramidal neurons. Many cellular subtypes within the cortex express AMPA and NMDA receptors, including pyramidal neurons but also GABAergic interneurons (e.g., Cox and Racca, 2013; Le Magueresse and Monyer, 2013), and thus the effects that such an increase in ionotropic glutamate neurotransmission would have on the overall behavior of frontal cortex output cells remains unknown. Moreover, the cellular subtypes that express Arc mRNA after stress, in relation to a cognitive task or after 5-HT_2A_ receptor activation have not yet been determined.

Another mechanistic study demonstrated that administration of 8 mg/kg DOI significantly increased cortical expression in rats or wild type mice, but not in mice with a conditional knockout of the gene encoding for BDNF (Benekareddy et al., 2013). These data could indicate that BDNF signaling through TrkB receptors is necessary for Arc expression stimulated by 5-HT_2_ receptor activation. However, another possibility is that the attenuation of TrkB receptor activation suppresses a common intracellular signaling pathway. Given that 5-HT_2_ receptors and TrkB receptors both act to increase intracellular Ca^2+^ concentrations, it is possible that the attenuated Arc expression seen in BDNF KO mice is due to a reduction in intracellular Ca^2+^ signaling cascades. If true, then it is also possible that reduction of BDNF signaling through TrkB receptors would also abrogate Arc expression stimulated through AMPA, NMDA, or group 1 mGlu receptors.

Unfortunately, the effects of 5-HT_2_ receptor mechanisms on cognitive functions are not as well studied (Table 4). The available evidence paints a mixed picture of the effects of 5-HT_2_ receptor biologies on cognitive performance. Performance in the spontaneous alternation task is not altered by DOI administration at 1 mg/kg (Seibell et al., 2003), which is a dose that significantly increases Arc expression on its own in cortical regions (Pei et al., 2004). Similarly, performance in a modified version of the water maze was not altered by DOI administration at 0.1–0.25 mg/kg (Kant et al., 1998). Evidence on the effect of 5-HT_2A/2C_ receptor stimulation on autoshaping performance is also mixed, with one study showing a DOI-induced impairment at doses ranging from 0.1 to 1 mg/kg (Meneses, 2007), and another showing a DOI-induced improvement at 0.1 mg/kg (Meneses et al., 1997). Similarly, the effects of 5-HT_2_ receptor antagonists on cognitive function seem to be mixed. The non-selective 5-HT_2_ receptor antagonist ketanserin was found to improve autoshaping performance at an extremely low dose of 0.001 mg/kg, which is likely not related to 5-HT_2_ receptors considering the binding affinity for this receptor, while the 5-HT_2A_ receptor selective antagonist M100907 had no effect at doses up to 3 mg/kg (Meneses et al., 1997). Additionally, M100907 at 0.08 mg/kg and the 5-HT_2C_ receptor selective antagonist SB242084 at 0.5 mg/kg had no effect on performance in the delayed alternation task (Papakosta et al., 2013), although it is not clear that these doses should be considered pharmacologically active.

Taken together, the available data suggest that there is some dissonance between the consistent 5-HT_2_ receptor-mediated increases in Arc expression, and the mixed effects of similar doses on cognitive function. Some of this variance may be due to the regionally selective nature of 5-HT_2A_ receptor-stimulated Arc expression, which was most strongly present in the cortex, whereas some of the cognitive tasks evaluated in these studies were dependent on hippocampal function, where DOI had no effects on Arc expression. Unfortunately, we were not able to identify any studies that investigated clearly cortex-dependent cognitive tasks, such as the attentional set shifting task, and thus it is difficult to clearly evaluate the relationship between 5-HT_2_ receptor effects on Arc expression and cognitive function.

### Other serotonergic receptors

Unfortunately, there is a paucity of information on the manner with which other serotonergic receptors modulate Arc expression. Due to the limited scope of the available data, we have chosen to briefly review the effects of these serotonin targets in a single section and not to examine the effects of these receptors on cognitive function.

5-HT_4_ receptors are stimulatory GPCRs for which there is generally little accumulated knowledge. Activation of 5-HT_4_ receptors increases neuronal activity via Gs-mediated increases in protein kinase A (PKA) activity (reviewed in Pehrson and Sanchez, 2015). A study by Eriksson et al. (2012) in Flinders Sensitive Line rats demonstrated that acute administration of 1 mg/kg of the 5-HT_4_ receptor partial agonist RS67333 induced a significant increase in Arc mRNA expression that was observed selectively in the CA1 and DG subregions of the hippocampus, where 5-HT_4_ receptors are thought to be strongly expressed (Vilaró et al., 2005). Importantly, in this region 5-HT_4_ receptors are selectively expressed in excitatory pyramidal neurons and granule cells, and are not present in GABAergic interneurons (Peñas-Cazorla and Vilaró, 2014). Therefore, once again these increases in Arc expression probably reflect increased glutamatergic neurotransmission.

No changes in Arc expression were observed due to RS67333 administration in the parahippocampal regions, somatosensory cortex or amygdala (Eriksson et al., 2012). These changes were blocked by administration of the MEK inhibitor PD184161. Whether these effects on Arc expression are truly due to 5-HT_4_ receptor activation is somewhat questionable, given that RS67333 is a partial agonist at these receptors, and importantly has a similar affinity for sigma 1 and 2 receptor sites (Eglen et al., 1995). Importantly, Eriksson et al. made no attempt to block the effects of RS67333 with a selective 5-HT_4_ receptor antagonist. However, a role for 5-HT_4_ receptor activation in Arc expression is supported by data suggesting that Gs-mediated increases in PKA activation also drive increased Arc expression (Bloomer et al., 2008).

Like 5-HT_4_ receptors, 5-HT_6_ receptors are stimulatory GPCRs that increase neuronal activity via Gs-mediated increases in PKA activity. 5-HT_6_ receptors are not well understood but are expressed in the neocortex by glia cells or pyramidal neurons, depending on the cortical layer in question (Marazziti et al., 2013). Administration of the putatively 5-HT_6_ receptor selective agonist LY586713 significantly increased the expression of Arc mRNA in the parietal cortex, cingulate, hippocampus, and dentate gyrus after acute administration (de Foubert et al., 2007). In addition, acute 5-HT_6_ receptor stimulation significantly increased the mRNA expression of BDNF in the hippocampus and dentate gyrus. The 5-HT_6_ receptor antagonist SB271046 had no effects on Arc expression in the hippocampus, dentate gyrus or parietal cortex on its own but significantly increased expression in the cingulate and orbital cortices (de Foubert et al., 2007). Interestingly, the acute effects of LY586713 in the hippocampus and parietal cortex were blocked by 5-HT_6_ receptor antagonism, but the increases observed in the cingulate and orbital cortices were maintained under these conditions (de Foubert et al., 2007).

Taken together, these data suggest that activation of 5-HT_4_ or 5-HT_6_ receptors, both of which are Gs-mediated stimulatory receptors, are capable of producing regional increases in Arc expression, although more work is necessary to replicate and confirm these data. However, especially in the case of 5-HT_4_ receptors, the available data appears to support the idea that Arc expression is associated with increased postsynaptic glutamate neurotransmission.

## Conclusion

This review has demonstrated that Arc is an effector IEG that plays an important role in dendritic plasticity and is closely associated with glutamate neurotransmission. This association with glutamate neurotransmission is bidirectional, since postsynaptic activation of stimulatory glutamate neurotransmission activates Arc expression, but Arc also plays a role in the trafficking and surface expression of AMPA receptors. Given recent data hinting at altered neuroplasticity in MDD, as well as data tying ketamine's fast-acting antidepressant effects to glutamate-dependent changes in dendritic plasticity (Li et al., 2010, 2011), Arc may represent an important mechanism for study in preclinical models of depression.

The data that are currently available from preclinical MDD models suggests that experimental treatments capable of producing a depression-like phenotype, such as chronic stress, induces an altered balance of Arc expression featuring suppressed expression in cortical regions and increases in the amygdala. Importantly, these changes in Arc stimulation appear to mirror stress-induced changes in glutamate neurotransmission. Additionally, known antidepressant treatments act to increase Arc expression, especially in cortical regions, and these antidepressant-induced effects on Arc expression share a similar timeline with the efficacy lag observed with these treatments. Furthermore, regional changes in Arc expression appear to predict the effects that experimental manipulations such as stress will have on cognitive function, with the general theme that increased Arc expression in a given area is associated with improved performance in cognitive tasks dependent on that area.

However, there are some caveats to consider. There is a host of clinical and preclinical evidence highlighting an important role for hippocampal neuroplasticity in MDD, but Arc expression generally does not appear to be altered in the hippocampus after either chronic stress or antidepressant treatments. These data would suggest that if hippocampal neuroplasticity is altered in these models, then it may be through Arc-independent mechanisms. Additionally, it is not possible at this time to conduct a complete evaluation of the relationship between cognitive function and pharmacological strategies for increasing Arc expression, which could include activation of 5-HT_2A_, 5-HT_4_, and 5-HT_6_ receptors, given the paucity of data available either for the effects of these receptor systems on Arc expression or cognitive function. However, the data that is available on these relationships does not universally support the simplistic notion that increased Arc expression is tied to improved cognitive function. Moreover, this idea is reinforced by observations that some effective treatments for mood symptoms that are known to increase Arc expression, for example NMDA receptor antagonists like ketamine or electroconvulsive stimulation (Larsen et al., 2005; Dyrvig et al., 2012; de Bartolomeis et al., 2013), are also known to impair cognitive function (Moscrip et al., 2004; Nikiforuk and Popik, 2014).

Thus, Arc appears to be a molecular target of some interest for the study of MDD and MDD-associated cognitive dysfunction. However there is much that remains to be done. Speaking generally, there has been little detailed study of how stress or antidepressants alter Arc expression in cellular sub-compartments such as the dendritic and nuclear regions. Given information on the role of nuclear translocation of Arc in reduced AMPA receptor expression, it is possible that Arc is in part responsible for some of the reductions in postsynaptic glutamate neurotransmission observed after chronic stress, and thus could play a role in driving cognitive impairment. Additionally, there has been essentially no work done to investigate what kind of cells, for example cortical pyramidal neurons vs. GABAergic interneurons, express Arc after manipulations such as stress or antidepressant treatment. Moreover, increasing the depth of our understanding of Arc expression in experimental contexts such as these may provide more clues on how the behavior of neural networks in the cortex and amygdala are altered in MDD, and how Arc expression is related to cognitive function.

### Conflict of interest statement

The authors declare that the research was conducted in the absence of any commercial or financial relationships that could be construed as a potential conflict of interest.
